# Evaluating the Safety and Efficacy of Ketamine as a Bronchodilator in Pediatric Patients With Acute Asthma Exacerbation: A Review

**DOI:** 10.7759/cureus.40789

**Published:** 2023-06-22

**Authors:** Abdullah S Binsaeedu, Deipthan Prabakar, Mohammed Ashkar, Cassie Joseph, Mohammed Alsabri

**Affiliations:** 1 College of Medicine, Alfaisal University College of Medicine, Riyadh, SAU; 2 Medical Intern, Government Kilpauk Medical College, Chennai, IND; 3 Lake Erie College of Medicine, Lake Erie College of Medicine, Erie, Pennsylvania, USA; 4 School of Science, Hampton University, Virginia, USA; 5 Paediatrics, Brookdale University Hospital Medical Center, Brooklyn, USA

**Keywords:** review., emergency department, pediatric, asthma, ketamine

## Abstract

Ketamine has emerged as a potential treatment option for pediatric patients with acute asthma exacerbation who do not respond to standard therapy. This review aims to evaluate the safety and efficacy of ketamine in this population and provide an overview of the current literature. A comprehensive search was conducted in PubMed and Google Scholar, resulting in the identification of four relevant studies. The studies demonstrated that ketamine administration led to improvements in respiratory parameters, including a decrease in clinical asthma scores (CASs) and respiratory rates, and an increase in peak expiratory flow and oxygen saturation. Ketamine infusion also showed promise in obviating the need for intubation in patients with severe wheezing due to bronchiolitis. The most common side effects observed were increased tracheobronchial secretions and hallucinations, which were manageable through discontinuation or additional medication. No significant changes in heart rate and blood pressure were reported, indicating hemodynamic stability. Long-term complications of ketamine use were minimal, with no reports of nightmares or dysphoria. In conclusion, ketamine shows potential as a bronchodilator for pediatric patients with acute asthma exacerbation, although further research is needed to fully evaluate its effectiveness and long-term effects. The use of ketamine should be considered in carefully selected cases and closely monitored for adverse events.

## Introduction and background

Asthma, a prevalent chronic respiratory disease among children globally, affects approximately six million children in the United States [1]. It is characterized by airway inflammation and manifests through symptoms such as cough, wheezing, shortness of breath, and chest pain/tightness [1]. Exacerbation of these symptoms indicates a worsening condition. Several factors have been identified as potential contributors to asthma exacerbations, encompassing inadequate asthma control, prior occurrences of severe exacerbations, exposure to viral and allergenic triggers, and a positive family history of asthma [2]. In 2019, the Centers for Disease Control and Prevention (CDC) reported over 700,000 emergency visits related to pediatric asthma, with 30% of children requiring further care after experiencing asthma exacerbations [3-4]. The primary pathophysiology of asthma involves airway hyperreactivity, increased vascular permeability, smooth muscle spasms, and release of inflammatory mediators [5].

Asthma management and treatment plans are tailored according to the severity of the patient's asthma. The initial approach typically involves the use of bronchodilators to induce relaxation of the bronchial smooth muscles, thereby improving breathing [6]. Beta-2-agonists, which are classified as short-acting beta-agonists (SABA) or long-acting beta-agonists (LABA), are commonly used to address bronchospasm. SABAs, such as albuterol, provide rapid relief and maintain their efficacy for up to 6 h, while LABAs are considered as a secondary therapy when SABAs fail to alleviate symptoms. Beta-agonists are generally preferred among the available treatment options [7]. Additionally, corticosteroids, like prednisone, are employed in asthma management to suppress airway inflammation. Anticholinergics, exemplified by ipratropium, exert their therapeutic effects by inhibiting involuntary movements and promoting bronchodilation. Research has demonstrated the effectiveness of ipratropium in managing exacerbations experienced by pediatric patients in the emergency department (ED) [7]. Furthermore, methylxanthines, including caffeine, theophylline, and aminophylline, facilitate the relaxation of the bronchial smooth muscles. Moreover, magnesium contributes to reducing airway constriction [7]. While asthma is a chronic respiratory condition without a cure, proper management involving appropriate combinations of medications can effectively control asthma exacerbations.

Ketamine, introduced for clinical use in the 1970s, is a dissociative anesthetic with diverse applications in the ED [8]. Its primary application is as a sedative during brief pediatric procedures and has shown therapeutic effects in patients presenting with status asthmaticus [2]. When traditional therapies fail to yield a response in pediatric patients, ketamine becomes the subsequent line of treatment [2]. Several characteristics make ketamine an excellent candidate for treating exacerbations: it exhibits a rapid onset of action within seconds and can be administered intramuscularly (IM), intravenously (IV), or intranasally [9]. Due to its high bioavailability and the low body mass of pediatric patients, IV or IM injections rapidly metabolize, allowing for higher dosing with continuous infusion [9]. Studies have demonstrated the benefits of continuous ketamine infusion in mechanically ventilated children with bronchospasm, including a decrease in the need for mechanical ventilation [5]. Ketamine also improves pulmonary compliance, and reduces airway resistance and bronchospasm, while preserving the airway reflexes of patients [5].

Therefore, this review aims to assess the safety and efficacy of ketamine in pediatric patients presenting with acute asthma exacerbation in the ED and provide an overview of the current literature on the subject.

## Review

Methodology

A comprehensive search was conducted in various databases, including PubMed and Google Scholar, to identify studies published from 1995 to 2023 that evaluated the effectiveness of ketamine as a bronchodilator in children with status asthmaticus. The search was restricted to studies published in English, and the most recent search was conducted in February 2023. The keywords used for the search included "ketamine," "status asthmaticus," "asthma," "bronchospasm," "bronchodilator," "children," "mechanical ventilation," and "invasive ventilation." The PubMed search yielded 91 articles, and the Google Scholar search yielded an additional 3230 articles.

To identify the relevant studies, a manual review of the bibliographies of the retrieved articles was performed based on pre-specified inclusion and exclusion criteria. The outcomes of interest were the effects of ketamine on respiratory parameters and vital signs, clinical status, need for invasive ventilation, adverse effects, and mortality.

The inclusion criteria for this review included primary articles, including randomized controlled trials (RCTs) and cohort studies that evaluated the role and effectiveness of ketamine as a bronchodilator in patients aged 2-≤ 21 years with status asthmaticus. Reference lists and bibliographies of eligible peer-reviewed articles were also searched for relevant material.

**Figure 1:**
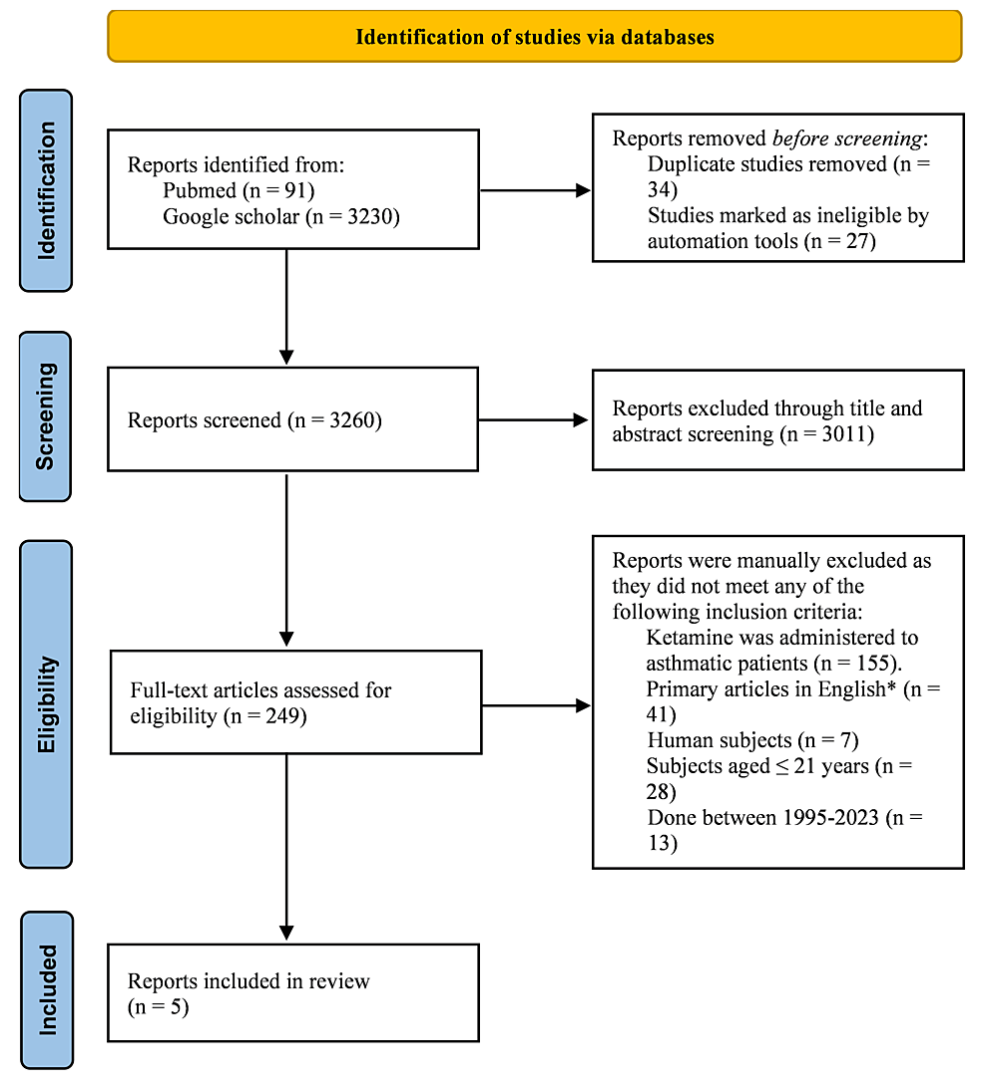
PRISMA flowchart. *Including randomized controlled trials (RCTs), control studies, and cohort studies PRISMA, Preferred Reporting Items for Systematic Reviews and Meta-Analyses

On the other hand, control trials involving animal subjects, case reports, case series, articles published before 1995, and non-English literature were excluded. Studies focused on ketamine use in the adult population (age above 21 years) and publications on ketamine for alternative treatments other than for asthmatic patients were also excluded. After reviewing the abstracts and full texts of relevant studies, studies not fulfilling the review's objective were excluded, leaving four to five articles for literature analysis (Figure 1).

Results

After an extensive search of PubMed and Google Scholar, we identified five articles that met our inclusion criteria. These studies, selected from an initial pool of 3321 articles, provided the most relevant insights regarding the efficacy of ketamine as a bronchodilator for patients aged ≤ 21 years with status asthmatics (Table 1). In the following section, we present a comprehensive summary of the methods and findings from each article. This summary covers the impact of ketamine on bronchodilation, its potential to avoid intubation, its effect on hemodynamic stability, as well as short-term and long-term side effects, along with the corresponding management strategies.

**Table 1:** Summary of included studies. PaO2, partial pressure of oxygen in arterial blood; FiO2, fraction of inspiratory oxygen concentration; SpO2, oxygen saturation; T0, prior to ketamine use; T1, 1 h after ketamine infusion; TW, when the ketamine was discontinued; CAS, clinical asthma score

Author	Year		Study design	Number of patients	Age	Mean age (years)	Ketamine dose	Duration	Intubation	Measures of improvement	Results	Adverse effects
Petrillo et al. [10]	2001		Prospective observational study	10	5-16 years	8	IV bolus 1 mg/kg followed by a continuous infusion of 0.75 mg/kg/h (12.5 mcg/kg/min).	1 h	No	CAS, peak expiratory flow, and vital signs	The study showed a significant decrease in the asthma score, improved peak expiratory flow, reduced respiratory rate, and enhanced oxygen saturation	Mild side effects (hallucinations, flushing, hypertension) in four patients were resolved with medication or discontinuation.
Youssef et al. [11]	1996		Retrospective chart review	17	5 months-17 years	6 +/- 5.7	IV bolus 2 mg/kg, followed by continuous infusions of 20-60 mcg/kg per minute (32 +/- 10 mcg/kg per minute)	40 h (12-96)	Yes	PaO2/FIO2, and dynamic compliance	PaO2/FIO2 ratio significantly increased after ketamine infusion at 1, 8, and 24 h. Dynamic compliance also improved.	One patient required glycopyrrolate for airway secretions, while another needed diazepam for ketamine-related hallucinations
Kshirsagar et al. [12]	2013		Prospective observational study	20	2 months-2 years	-	IV bolus 1 mg/kg followed by a continuous infusion of 10 mcg/kg/min (continuous infusion was given in 14 pts)	Average 15 h	No	Uyan score and SpO2	Uyan score significantly improved (T0: 13.14 ± 1.17, T1: 9.82 ± 1.21, TW: 0.5 ± 0.65). SpO2 also showed significant improvement (T0: 87.71% ± 2.05%, T1: 92.64% ± 2.46%, TW: 99.5% ± 0.51%).	None
Allen and Macias [13]	2005	Double-blinded, randomized, placebo-controlled trial		68	2-18 years	6.1 +/- 4.0	IV bolus 0.2 mg/kg followed by a 2-h ketamine infusion at 0.5 mg/kg/h	2 h	No	Pulmonary Index Score	No significant difference between groups	None
Tiwari et al. [14]	2016	Randomized, open-label, controlled trial		48	16 months-12 years	4	0.5 mg/kg bolus over 20 min, followed by continuous infusion of 0.6 mg/kg/h for 3 h	3 h	No	PRAM score	Similar primary outcomes were observed in both groups: ketamine (4.00 ± 1.25) and aminophylline (4.17 ± 1.68) with respect to PRAM score changes.	In the ketamine group, two patients experienced hypertension. Tachycardia was observed in both groups at enrollment and persisted after 3 h. Aside from one episode of vomiting in a patient from the ketamine group, no other adverse effects were reported in either group

Is Ketamine Effective as a Bronchodilator in Pediatric Status Asthmaticus?

In a prospective observational study conducted by Petrillo et al. [10], the effectiveness of ketamine in treating pediatric patients with status asthmaticus who were unresponsive to conventional therapy was evaluated. To this end, 10 children received a loading dose of 1 mg/kg intravenously for 15 min, followed by a continuous infusion at 0.75 mg/kg/h for 1 h. The results demonstrated a significant decrease in the median clinical asthma score (CAS) from 14.2 to 10.5 after 10 min of infusion, which further reduced to 9.5 after 1 h. Furthermore, the respiratory rate decreased by 25% 1-h post-infusion, and a significant improvement in peak expiratory flow was observed. Notably, oxygen saturation also exhibited a significant improvement 1 h after the infusion.

Similarly, in a retrospective analysis conducted by Youssef et al. [11], medical records of 17 mechanically ventilated children between 5 months and 17 years of age were analyzed to evaluate the effectiveness of ketamine infusion in treating refractory bronchospasm. It is worth noting that all patients had received conventional bronchodilatory therapy for over 24 h prior to starting ketamine treatment. Following a loading dose of 2 mg/kg ketamine administered intravenously, a continuous infusion of 20-60 mcg/kg per minute (mean dose 32+/-10 mcg/kg per minute) was initiated while maintaining the preexisting bronchodilatory regimen. The study assessed the PaO2/FIO2 ratio in all patients and dynamic compliance in 12 volume-preset mechanically ventilated patients. The findings revealed a significant increase in the PaO2/FIO2 ratio from 116+/-55 before ketamine to 174+/-82, 269+/-151, and 248+/-124 at 1, 8, and 24 h after the initiation of ketamine infusion (p < 0.0001). Additionally, dynamic compliance significantly increased from 5.78+/-2.8 cm3/cmH2O to 7.05+/-3.39, 7.29+/-3.37, and 8.58+/-3.69, respectively (p < 0.0001).

Moreover, in a study conducted by Kshirsagar et al. [12], 20 patients between 2 months and 2 years old suffering from severe wheezing due to bronchiolitis and unresponsive to standard treatment were administered a bolus dose of IV ketamine at 1 mg/kg after 48 h of humidified oxygen and nebulized adrenaline treatment. In cases where considerable improvement was not observed after 1 h, patients were given a continuous infusion of 10 mcg/kg/min. The Uyan score, which considers five crucial parameters such as respiratory rate, wheezing, retractions, nasal flaring, and general status, along with SpO2, was observed at three distinct time intervals: prior to ketamine use (T0), 1 h after ketamine infusion (T1), and when the ketamine was discontinued (TW). The results exhibited a gradual improvement in the Uyan score and a progressive rise in oxygen saturation, thereby eliminating the need for intubation and mechanical ventilation. Specifically, the mean Uyan score at T0, T1, and TW was 13.14 ± 1.17, 9.82 ± 1.21, and 0.5 ± 0.65, respectively, while the mean SpO2 at T0, T1, and TW was 87.71% ± 2.05%, 92.64% ± 2.46%, and 99.5% ± 0.51%, respectively.

However, a double-blinded, randomized, placebo-controlled study conducted by Allen and Macias [13] with 68 pediatric patients aged 2-18 years showed no significant difference in the mean pulmonary index score (PIS) between the intervention and control groups. Additionally, the mean decrease in PIS during the 2-h period was also not significantly different between the two groups. There were no significant differences noted in the degree of improvement in hypoxia, tachypnea, tachycardia, or blood pressure. The 33 patients in the intervention group received a ketamine infusion at 0.5 mg/kg/h after an IV bolus of ketamine at 0.2 mg/kg. The PIS was measured at 0, 30, 60, 90, and 120 min. However, two patients in the intervention group required IV terbutaline and increased doses of albuterol due to worsening bronchoconstriction, and they were subsequently admitted to the intensive care unit (ICU) for 3-4 days.

Furthermore, a study conducted by Tiwari et al. [14] analyzed the efficacy and safety of ketamine compared to aminophylline in 48 pediatric patients who responded poorly to standard therapy. A pediatric respiratory assessment measure (PRAM) score was used to measure a patient’s eligibility for the study as well as compare the effects of ketamine and aminophylline in refractory cases. The PRAM scores were measured every 30 min. Patients with PRAM scores >5 after 2 h of standard therapy were included in the study. Some 24 patients were administered ketamine at a dose of 0.5 mg/kg bolus over 20 min, followed by continuous infusion of 0.6 mg/kg/h for 3 h. Another 24 patients were administered aminophylline using 5 mg/kg bolus over 20 min followed by the continuous infusion of 0.9 mg/kg/h for 3 h. The PRAM score decreased significantly from enrolment to 3 h of intervention (7.88 ± 1.61 vs. 3.83 ± 1.86; mean difference 4.04; 95% confidence interval [CI] 3.61,4.47; p = 0.000). The PRAM score decreased significantly in both the ketamine (from 7.71 ± 1.68 to 3.79 ± 1.84; mean difference 3.92; 95% CI 3.39,4.44; p = 0.000) and aminophylline (from 8.04 ± 1.55 to 3.88 ± 1.92; mean difference 4.12; 95% CI 3.46,4.87; p = 0.000) groups. Overall, the PRAM score was similar between the groups (3.79 ± 1.84 vs. 3.88 ± 1.92; mean difference 0.08; 95% CI −1.18,1.01; p = 0.879). Therefore, although there was no statistical difference identified in the ketamine and aminophylline groups, it was found that ketamine led to a significant reduction in PRAM scores in patients who responded poorly to standard therapy.

Does Ketamine Obviate the Need for Intubation?

The study conducted by Kshirsagar et al. [12] revealed that the administration of ketamine to patients with severe wheezing caused by bronchiolitis, who exhibited a lack of response to standard treatment, led to notable improvement. As a result, the necessity for intubation and mechanical ventilation was eliminated. Tiwari et al. [14] also noted that patients who responded poorly to standard therapy improved after ketamine administration and did not require intubation.

Ketamine Side Effects and Management

The side effects observed during the studies encompassed hypertension, visual hallucinations, skin flushing, and increased tracheobronchial secretions leading to aspiration.

In Petrillo et al.'s [10] study, various side effects were reported, including hypertension, visual hallucinations, and skin flushing. One patient exhibited significant hypertension (149/105) following the initial bolus, which resolved within 10 min upon discontinuation of the infusion. Another patient experienced visual hallucinations approximately 30 min after starting the ketamine infusion. Despite midazolam administration, the hallucinations persisted but resolved once the infusion was discontinued. Similarly, a second child experienced visual hallucinations that successfully subsided with midazolam, allowing the continuation of ketamine therapy. Additionally, one patient developed diffuse skin flushing after approximately 20 min of infusion, without any associated cardiorespiratory changes. The flushing resolved when the infusion was discontinued. Importantly, all these reactions were effectively managed through the use of benzodiazepines or discontinuation of the drip.

Kshirsagar et al. [12] reported self-limiting side effects, such as increased secretions observed in all patients, tachycardia, and aspiration detected in two patients.

In Youssef et al.’s [11] study, adverse effects were observed in two patients. One patient experienced excessive tracheobronchial secretions, which were effectively treated with a daily dose of glycopyrrolate at 0.4 mg divided into four doses. Another patient exhibited hallucinations as an emergence phenomenon, successfully managed by administering a diazepam bolus of 0.2 mg/kg.

Conversely, Allen and Macias [13] reported no significant adverse effects, including dysphoria, laryngospasm, salivation, or nystagmus.

Tiwari et al. [14] noted that two patients in the ketamine group developed hypertension. At enrolment and after 3 h of intervention, all patients were normotensive. There were no other adverse effects in both the groups except for one episode of vomiting in one patient from the ketamine group.

None of the aforementioned studies reported any life-threatening adverse effects. The most frequently observed side effects included increased tracheobronchial secretions and hallucinations. In most cases, discontinuing the ketamine infusion or administering diazepam successfully resolved the hallucinations. Meanwhile, increased secretions were typically managed through the use of glycopyrrolate.

Ketamine Safety and Hemodynamic Stability

None of the above-mentioned studies reported significant changes in heart rate and blood pressure during the course of the study. In Petrillo et al.'s study [10], one patient experienced high blood pressure (149/105), which promptly resolved upon discontinuation of the infusion. In Tiwari et al.’s study [14], two patients given ketamine developed hypertension but both became normotensive by the end of the infusion. Similarly, Kshirsagar et al. [12] reported self-limiting tachycardia in some patients. Notably, Youssef et al. [11] found no significant alterations in heart rate or blood pressure among the patients studied, thereby eliminating the need for treatment.

 *Long-Term Complications of Ketamine*

In order to assess long-term complications, Allen and Macias [13] established contact with the families of patients who had received ketamine infusion, conducting telephone follow-ups. Encouragingly, none of the families reported nightmares, dysphoria, or any long-term abnormal changes in behaviors.

Discussion

Is Ketamine Effective as a Bronchodilator in Pediatric Status Asthmaticus?

The objective of this investigation is to evaluate the efficacy of ketamine in the treatment of pediatric patients suffering from status asthmaticus or refractory bronchospasm. Several studies, conducted by Petrillo et al. [10], Youssef et al. [11], Kshirsagar et al. [12], and Tiwari et al. [14] have demonstrated a positive impact of ketamine on asthmatic patients. These studies indicate improvements in oxygen saturation levels and clinical status, as measured by various rating scales. These findings are consistent with existing literature.

Goyal and Agrawal [5] conducted a comprehensive review that revealed the bronchodilator potential of ketamine when administered to asthmatic patients. The review highlighted its ability to induce airway relaxation by targeting various receptors and inflammatory cascades. Fischer [15], in a case report, along with Rock et al. [16] and Denmark et al. [17] in their respective case series, provide support to the discussion by demonstrating that the use of ketamine effectively relieves bronchospasm in pediatric patients who do not respond to conventional therapies. An extensive review conducted by Hendaus et al. [18], encompassing articles published from 1918 to June 2015 revealed contradictory findings regarding the use of ketamine for acute severe asthma in children. However, the review stated that given the drug’s notable safety profile, ketamine could be considered a potentially suitable approach for managing acute severe asthma in children who do not exhibit a positive response to conventional therapy. Based on these findings, it can be concluded that ketamine shows promise and effectiveness as a bronchodilator.

Does Ketamine Obviate the Need for Intubation?

The study conducted by Kshirsagar et al. [12] and Tiwari et al. [14] established the efficacy of ketamine administration in patients who were unresponsive to standard treatment, resulting in a significant improvement that led to the avoidance of intubation and mechanical ventilation. This finding is substantiated by additional studies in the existing literature. For instance, Denmark et al. [17] demonstrated that IV ketamine administration has demonstrated promising potential as a temporary measure to mitigate the necessity of subjecting children to the potential hazards associated with mechanical ventilation during severe asthma exacerbations. Furthermore, the report by Strube and Hallam [19] described the successful treatment of a 13-year-old female patient with severe respiratory compromise, where improvement was observed following ketamine infusion, ultimately avoiding the need for intubation and mechanical ventilation. Collectively, these studies suggest that ketamine could serve as an effective intervention for preventing the requirement of mechanical ventilation in patients experiencing severe asthma exacerbations and bronchiolitis.

Ketamine Side Effects and Management

The purpose of this investigation is to evaluate the reported side effects of ketamine use in clinical settings. Previous studies by Petrillo et al. [10], Youssef et al. [11], Kshirsagar et al. [12], Allen and Macias [13], and Tiwari et al. [14] have identified some side effects associated with ketamine use. However, these side effects were typically mild and manageable with medications or discontinuation of the infusion. According to the study conducted by Mason et al. [20], adults who are administered ketamine may experience side effects such as hallucinations, delusions, nightmares, and emergent delirium. However, a randomized double-blind placebo-controlled trial shows that these occurrences are infrequent in children below the age of five years [21]. In a separate investigation conducted by White et al. [22], it was observed that ketamine displays sympathomimetic properties by enhancing central catecholamine activity. However, they concluded that these side effects are counteracted by the peripheral myocardial depressant effects of ketamine, which leads to an attenuated catecholamine response that is not problematic. Ketamine shows some potential as a bronchodilator for pediatric patients with refractory bronchospasm, owing to its mild and manageable side effects in children. However, the existing evidence remains insufficient to draw definitive conclusions. Therefore, in order to ensure the safety of ketamine and comprehensively assess its suitability as a treatment option, it is imperative to conduct a rigorous double-blind RCT.

Ketamine Safety and Hemodynamic Stability

The safety and hemodynamic stability of ketamine have been extensively investigated in the aforementioned studies. Petrillo et al. [10], Youssef et al. [11], Kshirsagar et al. [12], and Tiwari et al. [14] reported no significant alterations in heart rate and blood pressure throughout their respective studies. These findings are in line with other relevant literature. Migita et al. [23] demonstrated that ketamine maintained the hemodynamic stability of patients, along with their spontaneous respirations and airway reflexes. Additionally, Bali et al. [9] showcased ketamine-induced rapid and consistent anesthesia without compromising hemodynamic stability and respiratory reflexes. The sympathomimetic effect of ketamine plays a crucial role in sustaining cardiovascular stability, counterbalancing its direct negative inotropic action on the heart. Moreover, the study conducted by Green et al. [24] provided compelling evidence indicating that the safety margin of ketamine administration remains substantial, even when non-anesthesiologists administer it in settings where basic mechanical monitoring is unavailable. In conclusion, the accumulated evidence indicates that ketamine is a safe and effective option for clinical use.

Long-Term Complications of Ketamine

Allen and Macias [13] conducted a study aimed at examining potential long-term complications associated with the use of ketamine in ED patients. The study did not observe any instances of nightmares, dysphoria, or long-term abnormal behavioral changes, suggesting that patients may tolerate the drug well in the long term without significant negative effects. However, Pearce et al. [25] discovered that nearly one in four children experienced substantial negative behavioral changes within 1-2 weeks after discharge from IV ketamine therapy. Furthermore, Meyers et al. [26] conducted a study demonstrating long-term hallucinations in two children following ketamine administration. The first child exhibited behavioral changes, including fearfulness towards traffic lights and recurring nightmares, over a 12-month period. The second child displayed biting behavior towards family members, which ceased after 1 year. However, the study acknowledged that these behavioral changes could be attributed to pre-existing personality issues rather than the administration of ketamine. Although the literature mentions these long-term complications, these studies are limited by the presence of multiple variables that can influence children's behavior and contribute to negative outcomes, including stress within the home environment and trauma resulting from the initial accident. Therefore, the definitive determination of ketamine's long-term safety remains elusive. A well-designed multicenter study with a larger sample size and extended follow-up periods are necessary to identify any potential short- or long-term complications associated with ketamine use in ED patients, particularly in those with asthma.

Strength and Limitations

The studies mentioned above have certain strengths and limitations that need to be considered. Petrillo et al.'s pilot study [10] found that adding ketamine to standard therapy improved respiratory symptoms in patients with status asthmaticus. However, the study was limited by a small sample size, a lack of randomization, and the need for a randomized, placebo-controlled trial to adequately determine the effects of ketamine. Allen and Macias's study [13] showed that ketamine, in addition to standard therapy, provided a significant improvement in the PIS for children with moderate to severe asthma exacerbations. However, the study was limited by the inability to study ketamine alone without albuterol due to ethical concerns, the subjectivity of the scoring scale used to detect changes in improvement, and the use of a convenience sample. Kshirsagar et al.'s study [12] found that ketamine was effective in treating bronchospasm in patients with wheezing due to bronchiolitis. However, the study is limited by its inability to test for respiratory syncytial virus due to the unavailability of the required test, and therefore, its inability to determine the complete efficacy of ketamine. Additionally, the need for a well-designed randomized control study in the pediatric age group is highlighted. Moreover, many of these studies including Youssef et al.'s study [11] had small sample sizes, making it difficult to establish the safety of certain treatment regimens. While Tiwari et al.’s study [14] reported comparable effectiveness between the two interventions, several limitations should be addressed to enhance its validity. These limitations include the absence of a placebo control group, lack of blinding for the treating team and patients, and the study's single-center nature. Additionally, the study did not perform viral studies on children below 2 years of age. Ketamine exhibits potential as a bronchodilator for pediatric patients experiencing refractory bronchospasm. Nevertheless, in order to establish its safety and assess its suitability as a treatment option, it is imperative to conduct well-designed RCTs with larger sample sizes. These trials should incorporate standardized criteria for evaluating improvements in respiratory symptoms and adverse events, with a specific emphasis on pediatric patients with asthma. By implementing these measures, we can obtain comprehensive insights into the safety and efficacy of ketamine within the pediatric population.

## Conclusions

Ketamine, a dissociative anesthetic and analgesic agent with bronchodilatory properties, has attracted attention as a potential therapeutic option for managing status asthmaticus. Nevertheless, the existing evidence on its effectiveness remains limited and controversial. While current findings do not support the routine use of ketamine in pediatric patients with status asthmaticus, our review highlights its potential as a future treatment option. This is attributed to its ability to increase arterial pressure, reduce bronchospasm, protect airways, decrease airway resistance, and optimize pulmonary compliance when administered. To establish the efficacy of ketamine in this specific population, there is a clear need for rigorous research, including large-scale multicenter RCTs. These trials should aim to determine optimal dosing and administration protocols, as well as thoroughly evaluate potential side effects.

## References

[1] Zahran HS, Bailey CM, Damon SA (2018). Vital signs: asthma in children - United States, 2001-2016. Morb Mortal Wkly Rep.

[2] Maddox RP, Seupaul RA (2014). Is ketamine effective for the management of acute asthma exacerbations in children?. Ann Emerg Med.

[3] (2023). Most Recent National Asthma Data [Internet]. Centers for Disease Control and Prevention. Centers for Disease Control and Prevention. https://www.cdc.gov/asthma/most_recent_national_asthma_data.htm.

[4] Smith SR, Strunk RC (1999). Acute asthma in the pediatric emergency department.. Pediatr Clin N Am.

[5] Goyal S, Agrawal A (2013). Ketamine in status asthmaticus: a review. Indian J Crit Care Med.

[6] Cazzola M, Segreti A, Matera MG (2010). Novel bronchodilators in asthma. Curr Opin Pulm Med.

[7] Rehder KJ (2017). Adjunct therapies for refractory status asthmaticus in children. Respir Care.

[8] Cromhout A (2003). Ketamine: its use in the emergency department. Emerg Med (Fremantle).

[9] Bali A, Dang AK, Gonzalez DA (2022). Clinical uses of ketamine in children: a narrative review. Cureus.

[10] Petrillo TM, Fortenberry JD, Linzer JF (2001). Emergency department use of ketamine in pediatric status asthmaticus. J Asthma.

[11] Youssef-Ahmed MZ, Silver P, Nimkoff L (1996). Continuous infusion of ketamine in mechanically ventilated children with refractory bronchospasm. Intensive Care Med.

[12] Kshirsagar V, Ahmed V, Colaco S (2013). Use of ketamine in refractory bronchospasm - a study of 20 cases. JKIMSU.

[13] Allen JY, Macias CG (2005). The efficacy of ketamine in pediatric emergency department patients who present with acute severe asthma. Ann Emerg Med.

[14] Tiwari A, Guglani V, Jat KR (2016). Ketamine versus aminophylline for acute asthma in children: a randomized, controlled trial. Ann Thorac Med.

[15] Fischer MM (1977). Ketamine hydrochloride in severe bronchospasm. Anaesthesia.

[16] Rock MJ, Reyes de la Rocha S, L'Hommedieu CS (1986). Use of ketamine in asthmatic children to treat respiratory failure refractory to conventional therapy. Crit Care Med.

[17] Denmark TK, Crane HA, Brown L (2006). Ketamine to avoid mechanical ventilation in severe pediatric asthma. J Emerg Med.

[18] Hendaus MA, Jomha FA, Alhammadi AH (2016). Is ketamine a lifesaving agent in childhood acute severe asthma?. Ther Clin Risk Manag.

[19] Strube PJ, Hallam PL (1986). Ketamine by continuous infusion in status asthmaticus. Anaesthesia.

[20] Mason KP, Padua H, Fontaine PJ (2009). Radiologist-supervised ketamine sedation for solid organ biopsies in children and adolescents. Am J Roentgenol.

[21] Sherwin TS, Green SM, Khan A (2000). Does adjunctive midazolam reduce recovery agitation after ketamine sedation for pediatric procedures? A randomized, double-blind, placebo-controlled trial. Ann Emerg Med.

[22] White JM, Ryan CF (1996). Pharmacological properties of ketamine. Drug Alcohol Rev.

[23] Migita RT, Klein EJ, Garrison MM (2006). Sedation and analgesia for pediatric fracture reduction in the emergency department: a systematic review. Arch Pediatr Adolesc Med.

[24] Green SM, Clem KJ, Rothrock SG (1996). Ketamine safety profile in the developing world. Acad Emerg Med.

[25] Pearce JI, Brousseau DC, Yan K (2018). Behavioral changes in children after emergency department procedural sedation. Acad Emerg Med.

[26] Meyers EF, Charles P (1979). Prolonged adverse reactions to ketamine in children. Survey Anesthesiol.

